# Supplementary material for: Sharing a household with children and risk of COVID-19: a study of over 300 000 adults living in healthcare worker households in Scotland
Source: Arch Dis Child. 2021 Mar 18;106(12):1212–7. doi: 10.1136/archdischild-2021-321604 (PMC7985971; doi:10.1136/archdischild-2021-321604)
Supplement: Supplementary data [file archdischild-2021-321604supp001.pdf]

Table S1 Comorbidity among adults living in healthcare worker households by number of young children in household

	0 children aged 0-11	1 child aged 0-11	2 children aged 0-11	3 or more children aged 0-11
Any comorbidity	33470 (13.87)	3883 (9.43)	1859 (7.82)	286 (7.43)
Ischaemic heart disease	4067 (1.69)	256 (0.62)	74 (0.31)	13 (0.34)
Other heart disease	7298 (3.02)	604 (1.47)	250 (1.05)	38 (0.99)
Other circulatory system diseases	4386 (1.82)	494 (1.20)	237 (1.00)	49 (1.27)
Advanced chronic kidney disease	224 (0.09)	30 (0.07)	14 (0.06)	0
Asthma and chronic lower respiratory disease	5311 (2.20)	745 (1.81)	394 (1.66)	65 (1.69)
Neurological disorders	1249 (0.52)	161 (0.39)	91 (0.38)	11 (0.29)
Decompensated liver disease	203 (0.08)	13 (0.03)	8 (0.03)	0
Any immunological condition	217 (0.09)	25 (0.06)	13 (0.05)	0
Malignant Neoplasms	7854 (3.26)	819 (1.99)	443 (1.86)	52 (1.35)
Disorders of oesophagus, stomach and duodenum	5707 (2.37)	676 (1.64)	325 (1.37)	50 (1.30)
Diabetes, type 1	1617 (0.67)	274 (0.67)	160 (0.67)	17 (0.44)
Diabetes, type 2	7660 (3.17)	618 (1.50)	205 (0.86)	32 (0.83)
Diabetes, unknown type	470 (0.19)	57 (0.14)	24 (0.10)	6 (0.16)

Wood R, *et al. Arch Dis Child* 2021; 106:1212–1217. doi: 10.1136/archdischild-2021-321604

Tables S2a to S2d Number of adults living in healthcare worker households according household composition

Table S2a Number of adults aged ≥18 by number of young children and children aged 12-17 in household

Number of children aged 12 to 17 in household	0 children aged 0-11	1 child aged 0-11	2 children aged 0-11	3 or more children aged 0-11
0 aged 12-17	208714 (86.51)	29855 (72.47)	21470 (90.27)	3559 (92.44)
1 aged 12 to 17	24359 (10.10)	9521 (23.11)	2051 (8.62)	215 (5.58)
2 aged 12 to 17	7596 (3.15)	1677 (4.07)	244 (1.03)	61 (1.58)
3+ aged 12 to 17	597 (0.25)	145 (0.35)	18 (0.08)	15 (0.39)

Table S2b Number of adults aged ≥18 by number of young children and adults aged 18 or older in household

Number of adults aged 18 or older in household	0 children aged 0-11	1 child aged 0-11	2 children aged 0-11	3 or more children aged 0-11
1 aged ≥18	39395 (16.33)	5026 (12.20)	2041 (8.58)	365 (9.48)
2 aged ≥18	78980 (32.74)	23042 (55.93)	17499 (73.58)	3273 (85.01)
3+ aged ≥18	122891 (50.94)	13130 (31.87)	4243 (17.84)	212 (5.51)

Table S2c Number of adults aged ≥18 by number of young children and adults aged 65 to 74 in household

Number of adults aged 65 to 74 in household	0 children aged 0-11	1 child aged 0-11	2 children aged 0-11	3 or more children aged 0-11
0 aged 65-74	219469 (90.97)	39659 (96.26)	23208 (97.58)	3797 (98.62)
1 aged 65-74	17701 (7.34)	1310 (3.18)	509 (2.14)	43 (1.12)
2 aged 65-74	3925 (1.63)	229 (0.56)	66 (0.28)	10 (0.26)
3+ aged 65-74	171 (0.07)	0	0	0

Table S2d Number of adults aged ≥18 by number of young children and adults aged 75 or older in household

Number of adults aged 75 or older in household	0 children aged 0-11	1 child aged 0-11	2 children aged 0-11	3 or more children aged 0-11
0 aged ≥75	231223 (95.84)	40226 (97.64)	23387 (98.33)	3817 (99.14)
1 aged ≥75	8290 (3.44)	827 (2.01)	361 (1.52)	17 (0.44)
2 aged ≥75	1674 (0.69)	145 (0.35)	35 (0.15)	16 (0.42)
3+ aged ≥75	79 (0.03)	0	0	0

Table S3 Risk and hazard ratios for COVID-19 requiring hospitalisation for adults living in patient-facing healthcare worker households* by number of young children in household.

	**0 children aged 0-11**	**1 child aged 0-11**	**2 children aged 0-11**	**3 or more children aged 0-11**	**Per child**
Events	272	41	17	1	-
N	137697	25461	15485	2627	-
Risk per 10,000	19.8	16.1	11.0	3.8	-
Unadjusted	1	0.82 (0.58-1.14)	0.57 (0.35-0.93)	0.20 (0.03-1.41)	0.75 (0.62-0.90)
Model 1	1	0.94 (0.67-1.32)	0.67 (0.41-1.12)	0.24 (0.03-1.69)	0.82 (0.68-0.99)
Model 2	1	0.97 (0.69-1.37)	0.74 (0.44-1.24)	0.26 (0.04-1.84)	0.85 (0.71-1.03)
Model 3	1	0.99 (0.70-1.39)	0.75 (0.45-1.25)	0.26 (0.04-1.87)	0.86 (0.71-1.03)
Model 4	1	1.03 (0.73-1.45)	0.82 (0.49-1.37)	0.29 (0.04-2.07)	0.89 (0.74-1.08)

Model fitting and covariates as per footnote of Table 2 in the main manuscript. * Households where at least one healthcare worker occupies a patient facing role.

Figure S1 – Causal diagram showing assumed role of covariates included in model 4 (Tables 2-4 of main manuscript)

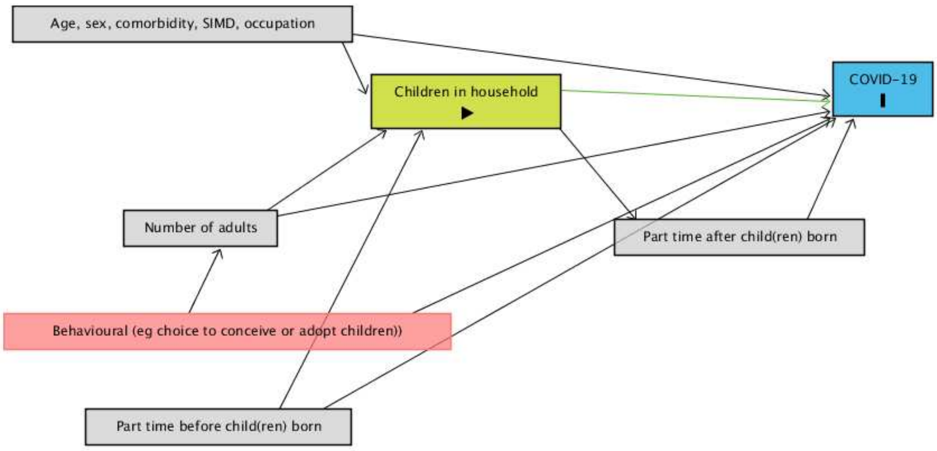

Green box indicates exposure, blue box outcome, grey boxes indicate variable/groups of variables conditioned on.

Wood R, *et al. Arch Dis Child* 2021; 106:1212–1217. doi: 10.1136/archdischild-2021-321604

Table S4 Hazard ratios for COVID-19 requiring hospitalisation for adults living in healthcare worker households by number of persons in household of different ages

	**Per child aged 0 to 4**	**Per child aged 5 to 11**	**Per child aged 12 to 17**	**Per adult aged 18 or above**
Unadjusted	0.63 (0.46-0.88)	1.03 (0.84-1.26)	0.91 (0.84-0.99)	0.84 (0.70-1.02)
Model 1	0.84 (0.61-1.15)	1.02 (0.84-1.25)	0.93 (0.85-1.01)	0.91 (0.74-1.11)
Model 2	0.84 (0.61-1.16)	1.04 (0.85-1.28)	1.00 (0.91-1.09)	0.92 (0.75-1.13)
Model 3	0.85 (0.62-1.17)	1.05 (0.86-1.29)	1.00 (0.92-1.09)	0.93 (0.76-1.14)
Model 4	0.88 (0.64-1.22)	1.07 (0.87-1.30)	1.00 (0.91-1.09)	0.96 (0.78-1.18)

Model specification and covariates as per footnote of Table 4.

Wood R, *et al. Arch Dis Child* 2021; 106:1212–1217. doi: 10.1136/archdischild-2021-321604

Table S5 Risks and hazard ratios for severe COVID-19 for adults living in healthcare worker households by number of young children in household

	**0 children aged 0-11**	**1 child aged 0-11**	**2 children aged 0-11**	**3 or more children aged 0-11**	**Per child**
N adults with severe COVID-19					-
Total N adults	241266	41198	23783	3850	-
Risk per 10,000	3.6	2.7	1.7	0.0	-
Unadjusted	1	0.75 (0.40-1.40)	0.48 (0.18-1.30)	-	0.68 (0.47-0.98)
Model 1	1	1.17 (0.62-2.23)	0.88 (0.32-2.37)	-	0.94 (0.66-1.33)
Model 2	1	1.25 (0.67-2.34)	0.87 (0.31-2.41)	-	0.95 (0.68-1.33)
Model 3	1	1.29 (0.69-2.42)	0.86 (0.31-2.41)	-	0.93 (0.67-1.30)
Model 4	1	1.36 (0.73-2.56)	0.97 (0.34-2.74)	-	1.01 (0.72-1.42)

Model fitting and covariates as per footnote of Table 2 in the main manuscript.

Table S6 Hazard ratios for severe COVID-19 for adults living in healthcare worker households by number of persons in household of different ages

	Per child aged 0 to 5	Per child aged 6 to 11	Per child aged 12 to 17	Per adult aged 18 or above
Unadjusted	0.57 (0.23-1.39)	0.54 (0.32-0.93)	1.22 (1.04-1.43)	0.74 (0.48-1.13)
Model 1	0.96 (0.41-2.22)	0.59 (0.35-1.00)	1.21 (1.02-1.44)	0.93 (0.61-1.43)
Model 2	0.90 (0.38-2.15)	0.59 (0.35-1.02)	1.20 (0.99-1.45)	0.97 (0.64-1.46)
Model 3	0.87 (0.36-2.08)	0.60 (0.35-1.02)	1.21 (1.01-1.45)	0.96 (0.64-1.45)
Model 4	0.95 (0.41-2.22)	0.60 (0.35-1.04)	1.21 (1.01-1.45)	1.04 (0.69-1.57)

Model specification and covariates as per footnote of Table 4.

Scottish household survey

As, we were unable to find any published studies providing empirical evidence to support or refute the claim that adults who shared a household with young children have restricted activity outside the home, we downloaded and analysed the 2018 Scottish Household Survey (which is available via the UK data Service at https://beta.ukdataservice.ac.uk/datacatalogue/series/series?id=2000048).

The 2018 Scottish Household Survey includes a travel diary where a "random adult" respondent in each household is asked to record all the activities they travelled to/from within the previous 24 hours. These activities are then assigned to standard categories. The Scottish Household Survey also records the number of children aged 0-11 and the number of children aged 0-4 in each household. This therefore provides a high-quality contemporaneous dataset for comparing the activities of households containing different numbers of children.

We restricted the data to households where the "random adult" was aged 25 to 44. We did so to avoid confounding without having to resort to regression modelling. Because we were interested solely in relative differences, we did not use the survey weighting variables which account for oversampling of different geographic regions. We compared the activities using both the original categorisations from the Scottish Household Survey and after collapsing these into broader categories relevant to SARS-CoV-2 exposure. In the supplementary appendix we provide both tables in full; below for convenience we have reproduced the collapsed-categories as well as selected rows showing the original categorisations.

Wood R, *et al. Arch Dis Child* 2021; 106:1212–1217. doi: 10.1136/archdischild-2021-321604

Table S7 Collapsed categories of activities. Number (%) of households where the "random adult" travelled to/from activities according to the number of children aged 0 to 11.

	0	1	2	3 or 4
Number of households	1085	458	421	80
Age random adult in household - mean (standard deviation)	33.8 (5.9)	35.7 (5.2)	35.8 (5)	35.05 (4.8%)
Community activities, indoor	362 (33.4%)	156 (34.1%)	147 (34.9%)	30 (37.5%)
Community activities, outdoor	5 (0.5%)	7 (1.5%)	6 (1.4%)	2 (2.5%)
Community activities, indoor or outdoor	41 (3.8%)	18 (3.9%)	18 (4.3%)	5 (6.3%)
Educational	33 (3%)	60 (13.1%)	79 (18.8%)	23 (28.8%)
Seeing friends	89 (8.2%)	21 (4.6%)	24 (5.7%)	1 (1.3%)
Home (journeying to or from)	210 (19.4%)	92 (20.1%)	83 (19.7%)	24 (30%)
Hospital	11 (1%)	6 (1.3%)	5 (1.2%)	1 (1.3%)
Other	141 (13%)	77 (16.8%)	69 (16.4%)	14 (17.5%)
Private	72 (6.6%)	19 (4.1%)	21 (5%)	8 (10%)
Relatives	106 (9.8%)	38 (8.3%)	30 (7.1%)	7 (8.8%)
Work	516 (47.6%)	192 (41.9%)	171 (40.6%)	28 (35%)

Table S8 Collapsed categories of activities. Number (%) of households where the "random adult" travelled to/from activities according to the number of children aged 0 to 4

	0	1	2 or 3
Number of households	1493	432	119
Age random adult in household - mean (standard deviation)	34.9 (5.8)	34.3 (4.9)	33.8 (4.9)
Community activities, indoor	504 (33.8%)	147 (34%)	44 (37%)
Community activities, outdoor	9 (0.6%)	6 (1.4%)	5 (4.2%)
Community activities, indoor or outdoor	60 (4%)	19 (4.4%)	3 (2.5%)
Educational	102 (6.8%)	64 (14.8%)	29 (24.4%)
Seeing friends	107 (7.2%)	22 (5.1%)	6 (5%)
Home (journeying to or from)	291 (19.5%)	88 (20.4%)	30 (25.2%)
Hospital	15 (1%)	6 (1.4%)	2 (1.7%)
Other	208 (13.9%)	81 (18.8%)	12 (10.1%)
Private	94 (6.3%)	15 (3.5%)	11 (9.2%)
Relatives	135 (9%)	36 (8.3%)	10 (8.4%)
Work	694 (46.5%)	170 (39.4%)	43 (36.1%)

Wood R, *et al. Arch Dis Child* 2021; 106:1212–1217. doi: 10.1136/archdischild-2021-321604

Table S9 Number (%) of households where the "random adult" travelled to/from activities according to the number of children aged 0 to 11

**Children aged 0 to 11**	**0**	**1**	**2**	**3or4**
**Number of households**	1085	458	421	80
**Age random adult in HH - mean (sd)**	33.8 (5.9)	35.7 (5.2)	35.8 (5)	35.05 (4.8)
**Other medical related eg. dentist, physio**	5 (0.5%)	7 (1.5%)	1 (0.2%)	1 (1.3%)
**Place of worship eg. church, mosque**	5 (0.5%)	8 (1.7%)	5 (1.2%)	1 (1.3%)
**The bank**	2 (0.2%)	1 (0.2%)	1 (0.2%)	0 (0%)
**The cinema**	8 (0.7%)	5 (1.1%)	2 (0.5%)	1 (1.3%)
**The Council offices**	2 (0.2%)	1 (0.2%)	0 (0%)	0 (0%)
**The Doctors**	6 (0.6%)	6 (1.3%)	5 (1.2%)	2 (2.5%)
**The gym**	20 (1.8%)	12 (2.6%)	12 (2.9%)	3 (3.8%)
**The pub**	22 (2%)	3 (0.7%)	0 (0%)	2 (2.5%)
**The restaurant**	31 (2.9%)	13 (2.8%)	9 (2.1%)	1 (1.3%)
**The shops**	287 (26.5%)	119 (26%)	130 (30.9%)	24 (30%)
**The park**	5 (0.5%)	7 (1.5%)	6 (1.4%)	2 (2.5%)
**Another entertainment/public activity**	15 (1.4%)	10 (2.2%)	6 (1.4%)	2 (2.5%)
**Participating in sport/exercise**	26 (2.4%)	9 (2%)	12 (2.9%)	3 (3.8%)
**College**	0 (0%)	4 (0.9%)	1 (0.2%)	0 (0%)
**School**	0 (0%)	1 (0.2%)	1 (0.2%)	1 (1.3%)
**School/college/university**	24 (2.2%)	5 (1.1%)	2 (0.5%)	0 (0%)
**School/nursery**	8 (0.7%)	51 (11.1%)	77 (18.3%)	22 (27.5%)
**University**	1 (0.1%)	1 (0.2%)	0 (0%)	0 (0%)
**Your friends**	89 (8.2%)	21 (4.6%)	24 (5.7%)	1 (1.3%)
**Home**	210 (19.4%)	92 (20.1%)	83 (19.7%)	24 (30%)
**The Hospital**	11 (1%)	6 (1.3%)	5 (1.2%)	1 (1.3%)
**Another personal appointment**	43 (4%)	21 (4.6%)	18 (4.3%)	4 (5%)
**Escort - other**	12 (1.1%)	22 (4.8%)	23 (5.5%)	4 (5%)
**On holiday**	2 (0.2%)	0 (0%)	0 (0%)	0 (0%)
**Other**	24 (2.2%)	6 (1.3%)	7 (1.7%)	3 (3.8%)
**Volunteering/caring**	8 (0.7%)	4 (0.9%)	2 (0.5%)	2 (2.5%)
**Your day trip**	13 (1.2%)	7 (1.5%)	16 (3.8%)	1 (1.3%)
**Your meeting**	46 (4.2%)	20 (4.4%)	13 (3.1%)	2 (2.5%)
**Take the dog out**	42 (3.9%)	5 (1.1%)	10 (2.4%)	4 (5%)
**Your cycle**	2 (0.2%)	2 (0.4%)	0 (0%)	0 (0%)
**Your drive**	3 (0.3%)	0 (0%)	1 (0.2%)	0 (0%)
**Your run**	5 (0.5%)	3 (0.7%)	5 (1.2%)	1 (1.3%)
**Your walk**	21 (1.9%)	10 (2.2%)	6 (1.4%)	3 (3.8%)
**Your relatives**	106 (9.8%)	38 (8.3%)	30 (7.1%)	7 (8.8%)
**Work**	516 (47.6%)	192 (41.9%)	171 (40.6%)	28 (35%)

Wood R, *et al. Arch Dis Child* 2021; 106:1212–1217. doi: 10.1136/archdischild-2021-321604

Table S10 Number (%) of households where the "random adult" travelled to/from activities according to the number of children aged 0 to 4

**Children aged 0 to 4**	**0**	**1**	**2or3**
**Number of households**	1493	432	119
**Age random adult in HH - mean (sd)**	34.9 (5.8)	34.3 (4.9)	33.8 (4.9%)
**Other medical related eg. dentist, physio**	9 (0.6%)	4 (0.9%)	1 (0.8%)
**Place of worship eg. church, mosque**	10 (0.7%)	9 (2.1%)	0 (0%)
**The bank**	4 (0.3%)	0 (0%)	0 (0%)
**The cinema**	14 (0.9%)	2 (0.5%)	0 (0%)
**The Council offices**	3 (0.2%)	0 (0%)	0 (0%)
**The Doctors**	14 (0.9%)	5 (1.2%)	0 (0%)
**The gym**	32 (2.1%)	11 (2.5%)	4 (3.4%)
**The pub**	24 (1.6%)	1 (0.2%)	2 (1.7%)
**The restaurant**	40 (2.7%)	10 (2.3%)	4 (3.4%)
**The shops**	398 (26.7%)	124 (28.7%)	38 (31.9%)
**The park**	9 (0.6%)	6 (1.4%)	5 (4.2%)
**Another entertainment/public activity**	21 (1.4%)	10 (2.3%)	2 (1.7%)
**Participating in sport/exercise**	39 (2.6%)	10 (2.3%)	1 (0.8%)
**College**	3 (0.2%)	2 (0.5%)	0 (0%)
**School**	2 (0.1%)	1 (0.2%)	0 (0%)
**School/college/university**	28 (1.9%)	2 (0.5%)	1 (0.8%)
**School/nursery**	69 (4.6%)	60 (13.9%)	29 (24.4%)
**University**	2 (0.1%)	0 (0%)	0 (0%)
**Your friends**	107 (7.2%)	22 (5.1%)	6 (5%)
**Home**	291 (19.5%)	88 (20.4%)	30 (25.2%)
**The Hospital**	15 (1%)	6 (1.4%)	2 (1.7%)
**Another personal appointment**	62 (4.2%)	23 (5.3%)	1 (0.8%)
**Escort - other**	36 (2.4%)	22 (5.1%)	3 (2.5%)
**On holiday**	2 (0.1%)	0 (0%)	0 (0%)
**Other**	28 (1.9%)	10 (2.3%)	2 (1.7%)
**Volunteering/caring**	11 (0.7%)	2 (0.5%)	3 (2.5%)
**Your day trip**	24 (1.6%)	12 (2.8%)	1 (0.8%)
**Your meeting**	58 (3.9%)	20 (4.6%)	3 (2.5%)
**Take the dog out**	54 (3.6%)	3 (0.7%)	4 (3.4%)
**Your cycle**	4 (0.3%)	0 (0%)	0 (0%)
**Your drive**	3 (0.2%)	0 (0%)	1 (0.8%)
**Your run**	11 (0.7%)	3 (0.7%)	0 (0%)
**Your walk**	25 (1.7%)	9 (2.1%)	6 (5%)
**Your relatives**	135 (9%)	36 (8.3%)	10 (8.4%)
**Work**	694 (46.5%)	170 (39.4%)	43 (36.1%)

Wood R, *et al. Arch Dis Child* 2021; 106:1212–1217. doi: 10.1136/archdischild-2021-321604
